# Sleep Quality and Fatigue during Exam Periods in University Students: Prevalence and Associated Factors

**DOI:** 10.3390/healthcare11172389

**Published:** 2023-08-25

**Authors:** Izolde Bouloukaki, Ioanna Tsiligianni, Giorgos Stathakis, Michail Fanaridis, Athina Koloi, Ekaterini Bakiri, Maria Moudatsaki, Eleptheria Pouladaki, Sophia Schiza

**Affiliations:** 1 Department of Social Medicine, School of Medicine, University of Crete, 71500 Heraklion, Greece; i.tsiligianni@uoc.gr; 2 Sleep Disorders Center, Department of Respiratory Medicine, School of Medicine, University of Crete, 71500 Heraklion, Greece; stathakisgg@gmail.com (G.S.); michfana@gmail.com (M.F.); athina_k45@hotmail.com (A.K.); aik.bakiri@gmail.com (E.B.); mariamoudatsaki@yahoo.gr (M.M.); elle.ria@hotmail.com (E.P.); schizas@uoc.gr (S.S.)

**Keywords:** sleep quality, fatigue, university students, exam period

## Abstract

The aim of our study was to assess university students’ sleep quality and fatigue before and during the academic exam period and identify potential associated factors. A Web-based survey was completed by 940 students of 20 different Tertiary Institutions including demographics, sleep habits, exercise, caffeine, tobacco, alcohol use, subjective sleep quality (Pittsburgh Sleep Quality Index—PSQI), and fatigue (Fatigue severity scale—FSS) at the beginning of the semester and during the examination period. During the exam period, PSQI (8.9 vs. 6.1, *p* < 0.001) and FSS scores (36.9 vs. 32.7, *p* < 0.001) were significantly elevated compared to the pre-exam period. An increase in the PSQI score was associated with age (β = 0.111, *p* = 0.011), presence of chronic disease (β = 0.914, *p* = 0.006), and depressive symptoms (β = 0.459, *p =* 0.001). An increase in the FSS score was associated with female gender (β = 1.658, *p* < 0.001), age, (β = 0.198, *p =* 0.010), increase in smoking (β = 1.7, *p =* 0.029), coffee/energy drinks consumption (β = 1.988, *p* < 0.001), decreased levels of physical exercise (β = 1.660, *p* < 0.001), and depressive symptoms (β = 2.526, *p* < 0.001). In conclusion, our findings indicate that exam periods have a negative impact on the sleep quality and fatigue levels of university students. Potential factors were identified that could contribute to the formulation of strategies for improved sleep quality and wellness.

## 1. Introduction

Adequate sleep duration has a critical role in promoting optimal physical health, immune function, mental health, and cognition [1]. According to consensus recommendations of the National Sleep Foundation and the American Academy of Sleep Medicine and Sleep Research Society guidelines, young adults should sleep 7 to 9 h per night on a regular basis for optimal sleep health [2,3]. Generally, deviating from the recommended sleep duration is associated with adverse health-related outcomes, including but not limited to poor attention, depression, obesity, and cardiovascular disease [4]. More specifically, sleep duration that deviates from the ideal range, either being excessively short or excessively long, appears to be linked to an elevated risk of all-cause mortality and cardiovascular events, with the risk being at its lowest when an individual sleeps for approximately 7 h per day [4]. Interestingly, earlier data suggests that inadequate sleep may be more concerning than excessive sleep in younger populations, as insufficient sleep durations were linked to poorer self-reported health in university students aged 17 to 30 years, while longer sleep durations were not related to self-reported health [5]. University students are considered as a population subset that is notably vulnerable to a shortened sleep duration and sleep disruptions [6,7]. Indeed, a young adult’s life is going through numerous transitions during their time at university in which students have reduced parental support, increased stress from academic loads and lifestyle, and irregularities in the sleep–wake cycle, all resulting in shortened and delayed sleep [8]. These sleep disruptions are of particular concern due to their negative impact on mental and physical well-being, as well as cognitive abilities that are crucial for students’ day-to-day performance and academic achievement [9,10,11]. Importantly, sleep quality in this population has been identified as the strongest predictor of well-being compared with physical activity, depression, and use of tobacco [12]. Attending university is also characterized by fatigue, which is often overlooked and also contributes to poor sleep [13].

Recent studies, representing different socio-cultural regions mainly from the US and China, have shown that sleep disturbances and dissatisfaction are particularly prevalent among university students, affecting 30 to 70% of this population [6,14,15,16,17,18,19,20,21,22,23]. During times of theorized greater stress, such as exams periods, students seem to demonstrate even worse sleep quality and less sleep than recommended [24]. However, these findings may not accurately represent sleep disorders rates among university students attending universities in Europe, which has distinct features associated with living arrangements, educational expenses, the application process, and facilities [25]. 

In Greece, research on sleep in university students remains scarce [26] and is mainly derived from studies during the COVID-19 pandemic [26,27,28]. Little is also known about how students’ sleep patterns change before and during the academic exam period [29,30]. To elaborate further, there is a scarcity of evidence pointing towards compromised sleep quality and quantity during exam periods, which may cause impaired daytime functioning [28,29,30]. Therefore, the aim of our study was to assess changes in Greek university students’ sleep quality and fatigue before (low stress) and during an academic exam (high stress) period and to identify possible associated factors.

## 2. Materials and Methods

### 2.1. Study Setting and Participants

A Web-based survey was conducted by university students of 20 different Tertiary Institutions (Medical/Health, Physics, Educational Sciences, Technical, Social sciences, Economic, etc.) in Crete, Greece, across 2 periods, at the beginning of the semester and during examination period in the academic year of 2018–2019 (before COVID-19 lockdown). The process of identifying and recruiting student participants involved two phases. The initial phase encompassed enlisting university professors and conveying the objectives of the current study on social media platforms. Subsequently, with the consent of their professors, university’s public e-mail board and social media platforms were used to send online anonymous survey links to students.

The students were asked to answer questions about demographics, sleep habits, exercise habits, caffeine, tobacco and alcohol use, hours of technology use (cell phones, tablets, laptop computers), obstructive sleep apnea (OSA) and insomnia symptoms, excessive daytime sleepiness, subjective sleep quality (using the Pittsburgh Sleep Quality Index—PSQI), and fatigue (Fatigue severity scale—FSS). Depressive mood, excessive daytime sleepiness, insomnia, and OSA symptoms were assessed by yes/no single item questions (“Have you felt depressed or sad/sleepy or had insomnia/OSA symptoms much of the time in the past month?”). 

All procedures were approved by the University of Crete Research Ethics Committee and all participants gave their informed consent to participate prior to both survey administrations, using a digital form.

### 2.2. Study Tools and Outcomes

#### 2.2.1. PSQI

A self-reported assessment of sleep was determined using the validated Greek version of the PSQI questionnaire, which is a standard instrument that has been validated as differentiating poor from good sleep [31,32]. The PSQI is a 19-item self-rated questionnaire that evaluates subjective sleep quality and quantity, sleep habits related to quality, and occurrence of sleep disturbances in adults over a 1-month interval. The 19 individual items are utilized in the generation of the following seven component scores: subjective sleep quality (one item), sleep latency (two items), sleep duration (one item), habitual sleep efficiency (three items), sleep disturbances (nine items), use of sleep medication (one item), and daytime dysfunction (two items). Each component score is equally weighted on a 0–3 scale, where 0 indicates no difficulty and 3 indicates severe difficulty. The aforementioned items, taken together, generate a score that reflects global subjective sleep quality, which varies between 0 and 21. The higher the score, the greater the negative impact on sleep quality. A global score of 6 or higher indicates poor sleep [33]. Previous studies have indicated adequate levels of internal consistency (Cronbach’s alphas 0.70–0.83), and construct validity on this questionnaire in various populations [32,33,34,35].

#### 2.2.2. FSS

In this scale, which is also translated and validated in Greek language [36,37], individuals rate their agreement (range, 1–7) with 9 statements concerning the severity, frequency, and impact of fatigue on daily life (physical functioning, exercise and work, and family or social life). Each item is rated on a 7-point Likert scale (1 ‘strongly disagree’ to 7 ‘strongly agree’). The FSS score is determined by calculating the average of all item scores. A total score of less than 36 is considered normal. A score above that limit (maximum score 63) is suggestive of a significant negative impact of fatigue on daily life activities [38]. FSS psychometric properties have been assessed in different populations indicating sufficient concurrent validity and internal consistency (Cronbach’s alphas 0.89–0.96) [36,37].

#### 2.2.3. Outcomes

The primary outcome of the study was to compare the absolute change in students’ global PSQI and FSS scores between a time of low stress (at the beginning of the semester) and a time of perceived high stress (academic exam period). Secondary outcomes involved identifying potential factors linked to baseline sleep quality, fatigue, and sleepiness, along with changes in sleep quality and fatigue among students.

### 2.3. Statistical Analysis

Our analysis was restricted to the subset of participants who gave responses in both the initial semester and exam period. Results are presented as mean ± standard deviation (SD) for continuous variables if normally distributed and as median (25–75th percentile) if not. Qualitative variables are presented as absolute number (percentage). To compare changes from the beginning of semester to exam period, the paired samples *t*-test (for normally distributed data) and the Wilcoxon Signed Rank test (for non-normally distributed data) were used. Changes of continuous variables from baseline to follow up were defined as baseline minus follow-up values. Factors associated with poor sleep quality and fatigue were analyzed with binomial logistic regression after adjustment for various potential explanatory variables, including age, gender, BMI, smoking status, presence of chronic disease, use of alcohol, caffeine, physical exercise, work, and use of technology. Multivariate linear regression analysis was used to examine any association of the previous potential confounders with changes in questionnaires scores (PSQI and FSS) during exam period. We checked multicollinearity among the predictors using collinearity statistics to ensure that collinearity between predictor variables was in the acceptable range as indicated by the tolerance value variance inflation factor. Internal consistency for PSQI and FSS was calculated with the Cronbach’s alpha (α) coefficient and item-total correlations. Spearman’s (rho) coefficients were used to calculate correlations between the questionnaire sub-scales. Test–retest reliability for the aforementioned questionnaires scores was explored with the intraclass correlation coefficient, ICC. Results were considered significant when *p* values were < 0.05. Data were analyzed using SPSS software (version 25, SPSS Inc., Chicago, IL, USA).

## 3. Results

### 3.1. Study Population

A total of 940 university students completed the questionnaire, of whom 60% were females (Table 1). Ages ranged from 17 to 48 years with a mean age of 21 years. Out of 940 participants, only a small fraction of 4 were above 40 years old and 9 were above 30 years old. Most of the participants were from Medical/Health (36%) and Physics (18%) universities. The majority of the participants were in the fourth year of education (50%) followed by the second (18%) and third (17%) years. Of note, 15% of students engaged in work and studies concurrently. Not a single participant reported having a family or young children.

Further sample characteristics including exercise habits, caffeine, tobacco and alcohol use, and hours of technology use are presented in Table 2. 

### 3.2. Sleep Patterns 

Sleep patterns of the surveyed students are depicted in Table 3, where most students (75%) report late bedtimes. No significant difference was noted in the number of students’ reported hours of sleep among all academic years (*p =* 0.095). Only 29 (3%) of the students reported frequently taking prescription medicine for insomnia.

According to the PSQI results, 554 (59%) out of 940 university students were classified as poor sleepers, a rate that remained similar across academic years (*p =* 0.299). On average, the PSQI global score of our sample was 6.1 ± 1.8 (Range: 1 to 14), which is above the cutoff for good sleepers (≤5), indicating that sleep quality was impaired. The ICC for the PSQI score was 0.717 and Cronbach’s α value was 0.739. The global PSQI score was significantly correlated with each component (*p* < 0.01). Item-total Spearman’s rho correlations ranged from 0.711 (component 1) to 0.838 (item 4). 

Impaired sleep quality was independently associated with the female gender (OR = 1.524, 95% CI 1.086–2.138; *p =* 0.015), younger age (OR = 0.922, 95% CI 0.866–0.981; *p =* 0.011), high alcohol consumption (OR = 2.095, 95% CI 1.023–4.290; *p =* 0.043), lack of physical activity (OR = 0.566, 95% CI 0.338–0.946; *p =* 0.030), presence of a chronic disease (OR = 2.695, 95% CI 1.542–4.711; *p* < 0.001), depressive symptoms (OR = 3.232, 95% CI 2.311–4.519; *p* < 0.001), sleepiness (OR = 9.893, 95% CI 5.163–18.956; *p* < 0.001), and fatigue (FSS ≥ 36) (OR = 1.550, 95% CI 1.094–2.195; *p =* 0.014).

### 3.3. Daytime Functioning

On average, the FSS total score of our sample was 32.7 ± 11.4 (Range: 9–62) and 365 out of 940 university students (39%) reported suffering from fatigue. The prevalence of fatigue remained similar across academic years (*p =* 0.754). The ICC for the FSS score was 0.845 and Cronbach’s α value was 0.868. The total FSS score was significantly correlated with each component (*p* < 0.01). Item-total Spearman’s rho correlations ranged from 0.529 (item 3) to 0.770 (item 6). 

Fatigue was independently associated with lack of physical activity (OR = 0.299, 95% CI 0.187–0.478; *p* < 0.001), presence of a chronic disease (OR = 1.619, 95% CI 1.080–2.426; *p =* 0.020), depressive (OR = 3.498, 95% CI 2.646–4.625; *p* < 0.001), and OSA symptoms (OR = 1.990, 95% CI 1.154–3.432; *p =* 0.013), sleepiness (OR = 1.911, 95% CI 1.333–2.739; *p* < 0.001), and impaired sleep quality (PSQI ≥ 6) (OR = 1.682, 95% CI 1.135–2.495; *p =* 0.010).

Regarding the frequency of excessive daytime sleepiness (two times or more per week), younger age (OR = 0.906, 95% CI 0.824–0.996; *p =* 0.040), presence of a chronic disease (OR = 2.071, 95% CI 1.145–3.747; *p =* 0.016), depressive symptoms (OR = 1.907, 95% CI 1.205–3.019; *p =* 0.006), presence of OSA symptoms (OR = 2.299, 95% CI 1.224–4.316; *p =* 0.010), and impaired sleep quality (OR = 14.565, 95% CI 6.166–34.042; *p* < 0.001) all significantly associated with more frequent reported sleepiness among students.

### 3.4. Changes in Sleep Quality and Fatigue during the Exam Period

During the exam period average sleep duration was significantly reduced (6.9 vs. 7.4, *p* < 0.001), 134 out of 242 (55%) smokers increased smoking, 489 out of 940 (52%) students increased coffee/energy drink consumption, 514 out of 940 (55%) decreased alcohol consumption and 474 (50%) decreased exercise. Insomnia symptoms, daytime sleepiness, and depressive symptoms were also more frequently reported in the exam period (Table 3).

PSQI global score was significantly elevated in the exam period compared to the pre-exam period (8.9 vs. 6.1, *p* < 0.001). Notably, all sub-scales of the PSQI contributed to the decline in sleep quality in the exam period (all *p* < 0.05). The increase in PSQI score (2.9 ± 1.6), which was similar in all years of education (*p =* 0.109), was independently associated with age (β = 0.111, *p =* 0.011), presence of chronic disease (β = 0.914, *p =* 0.006), worsening of FSS (β = 0.048, *p* < 0.001) depressive symptoms (β = 0.459, *p =* 0.001), and sleepiness (β = 0.601, *p* < 0.001). Furthermore, the prevalence of poor sleep quality (PSQI global score > 5) was also higher (98% vs. 59%) in this period.

FSS score was significantly elevated during the exam period compared to the pre-exam period (36.9 vs. 32.7, *p* < 0.001). Notably, all sub-scales of the FSS in the exam period were significantly elevated compared to the pre-exam period (all *p* < 0.001). This increase in FSS score (3.2 ± 6.4) was independently associated with the female gender (β = 1.658, *p* < 0.001), younger age, (β = 0.198, *p =* 0.010), increase in smoking (β = 1.7, *p =* 0.029), coffee/energy drinks consumption (β = 1.988, *p* < 0.001), decreased levels of physical exercise (β = 1.660, *p* < 0.001), depressive symptoms (β = 2.526, *p* < 0.001), sleepiness (β = 1867, *p* < 0.001), and worsening of PSQI (β = 0.754, *p* < 0.001). The prevalence of fatigue (FSS > 36) was also higher (50% vs. 39%, *p* < 0.001) in this period.

## 4. Discussion

The results of our study, obtained from students attending different Tertiary Institutions, showed that sleep quality and fatigue are frequent at the beginning of the semester and deteriorate during academic exam periods. Furthermore, this study provides a wide understanding of the factors that influence these observations during the demanding exam period. These factors include younger age, female gender, presence of chronic disease, decreased levels of physical exercise, depressive symptoms, and an increase in smoking and coffee/energy drinks consumption.

University students are recognized as a population group particularly affected by sleep problems and fatigue. In the present study, at the beginning of semester when stress would theoretically be low, about 60% of participants reported poor sleep quality with an average of 7.4 h of sleep per night, also indicating sleep deprivation among students. Increased autonomy, irregularities in the sleep–wake cycle, resulting from academic and social pressure at an age when the circadian rhythm is delayed and the central nervous system is still maturing, along with lifestyle and physical inactivity, increased caffeine and alcohol consumption may explain the poor sleep quality in this population [39,40,41,42]. Insomnia symptoms were also frequently reported, with similar [43] or higher rates compared to previous studies [44]. Potential factors that could contribute to poor sleep quality in our study were identified, such as being female, younger age, consuming high amounts of alcohol, not engaging in physical activity, and having a chronic disease, consistent with previous research [16,18,45,46,47,48]. Importantly, the presence of depressive symptoms was associated with an approximately threefold increase in the risk of reporting poor sleep. This association poses a significant concern for the mental well-being of students, given that sleep disruptions have been recognized as not only a risk factor for depression but also for other psychiatric disorders [49,50]. 

Academic responsibilities during university may also lead to fatigue, with concerning prevalence rates reported [51]. In our study, we found a fatigue prevalence of 39% in our university students at the beginning of semester, which is lower compared to a recent study (59.5%) [52] but higher compared to previous studies (16.7% and 13.7%) [53,54]. However, as a considerable proportion of our sample is from demanding disciplines like Medical/Health universities, a substantial prevalence of fatigue is anticipated. The level of fatigue was associated with the presence of chronic disease and depressive and OSA symptoms. At the same time, an inverse relationship was noted between fatigue and physical activity, in line with previous studies [52,55]. Consistent with previous research [52,55], poor sleep quality was also associated with fatigue, which could potentially have a more pronounced effect on university students’ academic performance and mental health. 

During periods of high stress, such as academic exam periods, university students are particularly affected by lower sleep duration and poor sleep quality [29,30]. In our study, shorter night sleeps during the exam period were reported compared with students’ typical routines and PSQI scores were significantly worse, with the prevalence of poor sleep quality increasing from 59 to 98%. Although there are several studies in the literature on the topic of sleep quality in university students, few studies have examined changes in university students’ sleep quality and fatigue across multiple time points (before and during an academic exam period) [24,29,30,56]. Based on recent research, university students exhibited negative outcomes, like deteriorating sleep quality [24,29,30], daytime dysfunction [29], and declining academic performance [30] during high-stress periods; however, the number of participants in previous studies was low (31–252 participants) [24,29,30]. In support of this, students who adhered to a regular sleep–wake routine in the 2 weeks preceding their end-of-semester exams tended to perform better academically [56]. Notably, in our cohort, we also highlighted a significant relationship between worsening of sleep quality and age, chronic disease, and depressive symptoms.

Further, we found that students who reported worsening sleep quality also had a higher risk of fatigue, the prevalence of which was also significantly elevated during the exam period, from 39% to 50%. The female gender, age, depressive symptoms, increase in smoking, coffee/energy drinks consumption, and lower physical activity levels were significant predictors of fatigue during exam periods. However, although the level of fatigue has been found previously to be associated with female gender, caffeine intake, physical activity, and sleep duration [52,57,58], these relationships in university students during exam periods are not documented. 

Considering that sleep disruption and fatigue potentially affect university students’ academic performance, getting sufficient quality sleep, especially before exams, may be associated with better academic performance, and lower odds of course failures [59]. Importantly, in a recent study, evaluating cognitive performance in medical students with visual and auditory evoked potentials, sleep deprivation during previous 2–3 nights before exam session and psychosomatic fatigue were found to be closely related to cognitive abilities, which in turn adversely affected academic achievements [60]. In support of this, it has been shown that university students who reported sleeping less than seven hours per night had a higher risk of exhaustion and low professional efficacy [30,61]. It is noteworthy that our findings are consistent with prior research indicating a strong correlation between poor sleep, excessive daytime sleepiness, and poor academic achievements [59,62]. Thus, it is crucial for universities to attempt to enhance students’ knowledge of how their lifestyle and sleeping behaviors can influence their academic performance. Such guidance along with recommendations for establishing good sleep hygiene would be valuable, particularly for first-year students and may enable students to improve sleep habits, not only in the university years but also in their later professional careers.

Our study has some limitations that deserve comments. First, the cross-sectional design of the current study precludes causal interpretations of the results. However, when examining the longitudinal portion of the research that examines the alterations in sleep quality, fatigue, excessive daytime sleepiness, and depression, it is possible to make some causal inferences about the impact of exam stress on the outcomes considered. Second, as this was a self-administered survey, it was prone to recall bias. Future studies including objective methods such as actigraphy or qualitative methods using semi-structured interviews and sleep diaries could obtain information that may have been overlooked due to the use of self-reported measures in the current study. Third, sleep quality and fatigue evaluation were taken from students studying in different institutions; therefore, factors such as academic demand and level of difficulty may potentially affect our results. Additionally, co-morbid mental health issues that could potentially impair sleep quality, such as anxiety, were not assessed and warrant consideration in future research. It should also be pointed out that this study did not account for participants who experienced a traumatic or highly stressful event between the two time points, such as a family member’s death, which could have undermined the validity of our results. Lastly, taking into account that the participants included lived in Crete (south of Greece), sleep quality and fatigue levels may be different in other regions of Greece. Consequently, the generalization of our conclusions to all Greek university students should be made with caution.

## 5. Conclusions

Our results indicate that sleep quality and fatigue are frequent and deteriorate in university students during academic exam periods. Potential factors associated with the risk of inadequate sleep quality and increased fatigue were identified, such as female gender, younger age, presence of chronic disease, increase in smoking, coffee/energy drinks consumption, decreased levels of physical exercise, and depressive symptoms, which may assist in prevention strategies to promote a better quality of sleep. Future studies are desirable to identify the mechanisms behind sleeping problems and fatigue during exam stress.

## Figures and Tables

**Table 1:** Characteristics of the participants (n = 940).

Characteristics	
**Age (years)**	21 ± 3
**Gender, male (%)**	371 (40%)
**BMI (kg/m^2^)**	23.7 ± 4.8
**BMI ≥ 30**	65 (7%)
**Current Smoking**	242 (26%)
**Type of education**	
Medical/Health	343 (36%)
Physics	166 (18%)
Educational Sciences	79 (8%)
Technical	82 (9%)
Social sciences	92 (10%)
Economic	130 (14%)
Other	41 (4%)
**Year of education**	
1st	136 (15%)
2nd	168 (18%)
3rd	162 (17%)
4th +	474 (50%)
**Working Students**	136 (15%)

Data are presented as mean values ± SD or median (25–75th percentile), unless otherwise indicated. BMI, body mass index.

**Table 2:** Exercise habits, caffeine, tobacco and alcohol use, and hours of technology use.

Characteristics	
**Alcohol consumption**	
Low (0–3 drinks/week)	718 (76%)
Moderate (4–8 drinks/week)	166 (18%)
High (≥9 drinks/week)	56 (6%)
**Coffee/energy drinks consumption**	
None	302 (32%)
Low (1–2 drinks/day)	557 (59%)
Moderate to high (>3 drinks/day)	81 (9%)
**Physical activity**	
None	328 (35%)
1–3/week	479 (51%)
>4/week	133 (14%)
**Hours of technology use**	
0–2 h/day	140 (15%)
2–5 h/day	449 (48/%)
5–8 h/day	230 (24%)
>8 h/day	121 (13%)

**Table 3:** Daytime symptoms, sleep patterns, and disorders of university students (n = 940).

Symptoms	Pre-Exam Period	Exam Period	*p*-Value
**Daytime symptoms**			
FSS	32.7 ± 11.4	36.9 ± 12.3	<0.001
FSS ≥ 36	365 (39%)	473 (50%)	<0.001
Excessive Daytime Sleepiness			
(2 times or more per week)	168 (18%)	222 (24%)	<0.001
Depressive mood	391 (42%)	547 (58%)	<0.001
**Sleep Characteristics**			
**Bedtime**			
Between 9 pm and 12 am	238 (25%)	230 (25%)	
After 12 am	702 (75%)	710 (75%)	0.643
**Wake up time**			
Between 5 and 8 am	219 (23%)	222 (23%)	
Between 8 and 11 am	485 (52%)	553 (59%)	
After 11 am	236 (25%)	165 (18%)	<0.001
**Sleep duration**	7.4 ± 1.3	6.9 ± 2.9	<0.001
**Frequent use of sleep medications**			
(2 times or more per week)	29 (3%)	41 (4%)	0.031
**PSQI**	6.1 ± 1.8	8.9 ± 1.9	<0.001
PSQI > 5	435 (59%)	919 (98%)	<0.001
**Insomnia symptoms**			
(2 times or more per week)	417 (44%)	505 (54%)	<0.001
**Obstructive Sleep Apnea symptoms**			
(2 times or more per week)	122 (13%)	131 (14%)	0.256

## Data Availability

The datasets generated during and/or analyzed during the current study are available from the corresponding author on reasonable request.
